# Attitudes and perceptions of final year medical students on sexual history taking from patients in Ogun state, Southwestern Nigeria

**DOI:** 10.4314/gmj.v55i2.6

**Published:** 2021-06

**Authors:** Oluseun O Adeko, Adekunle J Ariba, Akindele E Ladele

**Affiliations:** 1 Department of Family Medicine, Olabisi Onabanjo University Teaching Hospital, Sagamu, Nigeria; 2 Department of Family Medicine, Babcock University Teaching Hospital, Ilishan, Nigeria

**Keywords:** Sexual health, history taking, medical students, South-West Nigeria

## Abstract

**Background:**

An important aspect of sexual health is the ability to take a sexual history. Previous studies have shown that most medical students believed that sexual history taking is an important skill for future practice. Still, a majority reported inadequate, inconsistent or no training in this area.

**Objectives:**

To assess the attitudes of final year medical students on sexual history taking and perceptions of the training they received in medical school

**Design:**

A cross-sectional study using an online survey

**Participants:**

Consented and conveniently sampled 100 final year medical students.

**Results:**

The overall response rate was 74.6%, and the mean age of the respondents was 24.1±2.9 years. The majority (97%) of the students believed it is important for doctors to know how to take a sexual history. Still, only 31% admitted to finding it easy, with 57% of the students admitted to being comfortable taking a sexual history from adult patients. While 70% had exposure on simulated patients, just 54% have observed doctors taking sexual history during clinical rotations, mostly in Obstetrics and Gynaecology (97%) and Urology (60%) postings.

**Conclusions:**

Many final year medical students are interested in and appreciated the importance of sexual history taking, but they are not well grounded in many aspects of the topic. Despite the importance of sexual health, many students did not have enough exposure and training on the topic while still in medical schools. There is thus a need for a review of the curriculum of undergraduate medical education in Nigeria.

**Funding:**

Self-funded by the authors

## Introduction

Sexual health is an integral aspect of well-being and a strong determinant of quality of life in adolescence and adulthood. Early initiation of sexual activities increases the risk of Human Immunodeficiency Virus/Acquired Immunodeficiency Syndrome (HIV/AIDS), Sexually Transmitted Infections (STIs) and teenage pregnancy, among others.^1^ According to the Nigeria Demographic and Health Survey (DHS), the proportion of adolescents who have had sexual intercourse by age 18 years increased from 54% in 2013 to 57% in 2018.^2^ A study among secondary school students aged 10-19 years in Nigeria showed that the mean age of sexual debut was 13.0±2.3 years.^3^ Among the elderly sexual activities tend to decline with increasing age; however, recent studies have shown that many older adults engage in sexual activities, especially with the availability of effective medications for erectile dysfunction starting with Viagra® in 1989.^4^-^6^

In Nigeria, a study of 100 elderly persons with a mean age of 66.42 ±5.77 years found that 48% of the respondents still engaged in sexual activities and 71% considered it safe to do so.^7^

Given the importance of sexuality to overall health and well-being, it has been suggested that sexual health should be integrated with all aspects of patient care.^8^ To do this a medical doctor should be confident in taking a sexual history as a routine during clinical encounters. This has many benefits; the prevalence of HIV infection is still high, and 40% of new cases of infection in Africa are from Nigeria and are mainly acquired through sexual activities. 9 Taking sexual history as a routine will allow identification of risky sexual behaviours associated with HIV infection and serves as a primary prevention strategy.^10^ It also allows identification and treatment of various sexual dysfunctions and addresses other sexual concerns.

This is very important given the high prevalence of some of the sexual dysfunctions in the community. A population-based study of men aged 30 – 80 years in Ogbomoso, South-west Nigeria, found the prevalence of erectile dysfunction to be 58.9%.^11^ Some of these sexual dysfunctions may eventually be a pointer to serious health problems, thereby making sexual history taking a screening tool.^10^

Despite these benefits of taking a sexual history, studies have shown that doctors find it difficult to broach the subject of sex and do not routinely explore sexual practices of their patients.^11^, ^12^ Most primary care clinicians would not assess the patient's sexual health unless the patient brought up the issue.^13^ In contrast, most patients would like to discuss their sexual concerns with their doctors but prefer the doctors to take the initiative.^14^ It has also been found out that patients would prefer to discuss their sexual health issues with doctors they perceived as comfortable and skilled in this aspect.^15^ Studies have shown that most medical students believed that sexual history taking is an important skill for future practice. Still, the majority reported inadequate, inconsistent or no training in this area.^16^, ^17^ This had resulted in medical students being uncomfortable in taking a sexual history.^17^ When asked to take sexual history for the first time, a group of medical students reported their inability to frame sensitive questions and were worried about embarrassing themselves, making them omit certain topics entirely.^18^

After the undergraduate medical training, internship, and national service in Nigeria, the regulation permits independent private practice and even taking up a career in public hospital without further postgraduate training. For this reason, it is expected that the prospective doctors are well trained to be able to handle most common conditions including sexual health. In Nigeria, as in most other countries, there is no standardized curriculum on sexual health or on how to take a sexual history during the undergraduate medical training.^19^, ^20^ Medical students are however exposed to some aspects of sexual health during their rotations in various specialties. It is unknown how this will impact on their ability to take sexual history and handle sexual health issues when they start practicing as doctors. It was for this reason that this study was conducted to assess the readiness of the final year medical students in Nigeria to handle sexual health problems when they graduate by assessing their attitudes on sexual history taking and perceptions of the training they received in medical school.

## Methods

### Study design and population

This was an online cross-sectional survey of final year medical students of Olabisi Onabanjo University (OOU) and Babcock University (BU). OOU was established in 1982 as the first state government owned public university in Nigeria and the medical school became operational in 1986. BU is a pioneer private university in Nigeria established in 1999 and the medical school commenced operation in 2012. The two universities are located in Ogun state, Southwest Nigeria.

The study was conducted in May 2020 using online selfcompletion questionnaire. The participants in the study were the final year medical students in both schools. There were 60 and 74 final year medical students at OOU and BU respectively at the time the study was conducted. 40 students (66.7%) from OOU and 60 (81%) from BU participated in the study.

### Sampling method

The recruitment was by convenient sampling of all the 134 final year medical students in the two medical schools out of which 100 students gave informed consent and participated in the study.

### Study Instrument

The questionnaire was adapted with permission from the work of Ariffin *et al.*^21^ and consisted of two sections: demographic and survey questions sections. The students' attitudes to sexual history taking were assessed using 17 variables, while their perceptions were assessed with eight variables. The responses were in YES, NO and NEUTRAL format. The questionnaire was designed using the Survey Monkey tool and the link with a short description of the study was shared by WhatsApp and Email to all the final year medical students of the two institutions. The two approaches were used to share the link to increase the response rates.

### Statistical Analysis

This was done using IBM Statistical Package for Social Sciences (SPSS) version 20. Means with standard deviations and proportions were used to describe the demographic characteristics of the respondents. The responses were presented with tables using proportions.

### Ethical consideration

Approval for the study was given by the Health Research Ethics Committee of the Olabisi Onabanjo University Teaching Hospital with registration number NHREC/28/11/2017

## Results

### Demographic characteristics

Out of the 134 final year medical students invited to participate in this study, 40 and 60 students from OOU and BU responded. This gave a total of 100 final year medical students and an overall response rate of 74.6%. The mean age was 24.1 ± 2.9 years. As shown in Table 1 majority of the students (74%) were in the age group of 20-24 years, and female students constituted 64% of the respondents. None of the students was married.

**Table 1:** Demographic characteristics (n=100)

Variables	n(%)
**Age (years)**	
**20-24**	74(74)
**25-29**	22(22)
**≥30**	4(4)

**Gender**	
**Male**	36(36)
**Female**	64 (64)

**Religion**	
**Islam**	15(15)
**Christianity**	85(85)

**Medical school**	
**OOU**	40(40)
**BU**	60(60)

### Attitudes toward taking a sexual history

As shown in Table 2, 90% of the students were interested in learning about sexual health, and 97% thought that it is important for doctors to know how to take a sexual history. While 74% of the participants were comfortable taking a sexual history from adolescents, only 57% were comfortable with adult patients.

**Table 2:** Attitudes toward taking sexual history (n=100)

Variables	Yes %	No %	Neutral %
I am interested in learning about sexual health	90	1	9
I think that it is important for doctors to know how to take a sexual history	97	1	2
I feel that a nurse can take better sexual history than a Doctor	2	67	31
I feel comfortable in discussing sexual health problems with adult patients	57	25	16
I feel comfortable discussing sexual health problems with adolescent	74	16	10
I feel comfortable discussing sexual health problems with patients of opposite gender	51	27	22
I feel comfortable discussing sexual health problems with unmarried but sexually active Patients	72	13	15
I feel comfortable in asking patients about their sexual orientation e.g lesbianism	46	32	22
I feel comfortable in asking patients regarding their sexual practices e.g. “Are you sexually active?”, “Do you practice oral sex?”	61	25	14
I feel comfortable in taking a sexual history from patients who are uneasy in discussing sex	33	44	23
I feel that cultural differences are a barrier when discussing sexual health problems with patients	80	13	7
I feel that religious differences are a barrier when discussing sexual health problems with patients	73	17	10
I recognize my own limitations in discussing sexual health issues with patients	79	10	11
I have thought about how my own attitudes, beliefs and values may affect my discussion of sexual health issues with patients	67	29	4
I believe that it is important to maintain patient confidentiality	98	1	1
I feel that patients would like to discuss their sexual health problems with a senior doctor e.g. A consultant	75	11	14
I feel that taking a sexual history will take much of my time	13	78	9

Also, just 51% felt comfortable discussing sexual health problems with the opposite gender and fewer (41%) felt comfortable exploring the sexual orientation of patients. In terms of barriers to sexual history taking, culture (80%), limitation in expertise (79%), preference of the patients to discuss sexual health issues with a consultant (75%) and religion (73%) were predominantly identified.

### Perceptions of skills and training on sexual history taking

Table 3 shows the students' perceptions of their skills and their training on sexual history taking.

**Table 3:** Perception of training received on taking sexual history

Variables	Yes %	No %	Neutral %
I find taking sexual history easy	31	37	32
I have adequate skills to take a sexual history	38	33	29
I have adequate skills to put a patient at ease when discussing their sexual health issues	46	27	27
The training in my medical school prepares me to take a sexual history	51	18	21
I have enough exposure as a medical student to take a sexual history from a real patient	58	19	23
I have enough exposure as a medical student to take a sexual history from a simulated patient	70	15	15
I have enough exposure as a medical student observing Doctors take sexual history during clinical rotations	54	27	19
I feel that there is not enough training in the medical school on how to discuss sexual health problems with patients	51	30	19

**Figure 1:**
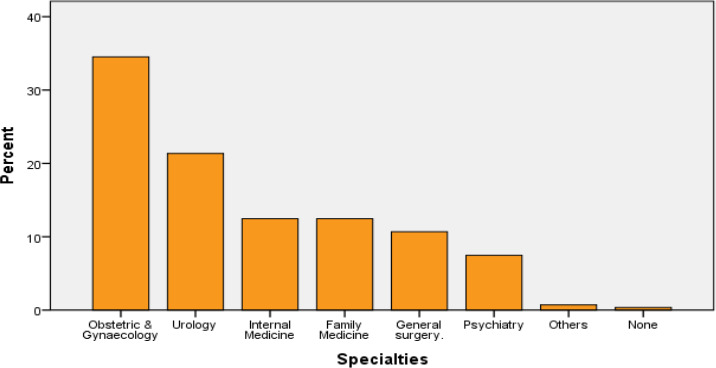
Exposure to sexual history taking in different specialties

It was only 31% of them who admitted to finding sexual history taking easy. On the adequacy of the training they received, 70% admitted to having enough exposure on simulated patients with fewer exposure in real-life clinical settings, such as taking a sexual history from real patients (58%) or observing Doctors taking sexual history (54%). Figure 1 shows the exposure of the students to sexual history taking according to the various specialties with the Obstetrics and Gynaecology and Urology being the specialties that offered the greatest exposures.

## Discussion

Findings from this study show that most of the students are interested in learning about sexual health, but they also recognized the need for doctors to know how to take a sexual history. These have been reported in similar studies among medical students and demonstrate a positive attitude towards sexual health.^21^,^22^ The study showed that 57% of the students admitted not being comfortable discussing sexual problems with adults while 74% were comfortable with adolescents. This might be related to the fact that 74% of the students were within 20-24 years. The age disparity with much older patients may limit effective communication given the sensitive nature of sexual health. Their being comfortable to engage adolescents on sexual health history is a positive finding as this attitude can facilitate early identification of risky sexual behaviours among younger persons. An increasing proportion of Nigerian adolescents are becoming sexually active, with many of them having had sexual intercourse by age 15 years.^3^ Also, only 51% of the students were comfortable discussing sexual health with the opposite gender. This has been demonstrated in other studies among doctors where it was identified as a barrier.

Other barriers identified by the majority of the students in this study were cultural differences and religion.

These have been established as barriers in previous studies among doctors.^14^, ^15^ Of note was that 75% of the students felt that patients prefer discussing sexual issues with Medical Consultants; this might reflect the expectations that Medical Consultants are likely to be much older, experienced and skilful in handling such sensitive issues.

In this study, only 38% rated their skills as adequate to take a sexual history, and 51% felt that there was not enough training on the subject. About half of the students admitted to having taken a sexual history directly from patients (58%) or even observed the process during clinical rotations (54%). These findings show that sexual health is not adequately taught at the undergraduate level.

This is not unique to Nigeria. A report from the United Kingdom government attributed why sexual health issues are not addressed during consultations to inadequate, patchy or absent training in the undergraduate curriculum.^23^ The scanty time allotted to teaching sexual history taking was shown in a survey of 22 medical students in the UK, which found the mean time allotted to it was 1.8 hours whereas students spent a mean of 6.8 hours in the Genito-Urinary Medicine clinics.^24^

As noted above, the barriers to exploring sexual health by the medical students are similar to those faced by practising doctors, particularly general practitioners, suggesting that the physicians' barriers are a carryover from medical schools. Since most medical students in Nigeria eventually become General Practitioners, the curriculum should be reviewed to ensure sufficient proficiency in sexual history before graduation.

Although various methods are used in clinical teachings, this study suggests that simulated patients were mainly used as 70% of the students admitted to having enough exposure by this method and just 54% admitted to observing doctors taking sexual history during consultations. This is not surprising as sexual history is not routinely taken during consultations. In a study of Obstetrics and Gynaecology Residents, only 5% admitted to taking sexual history frequently or always during consultations.^25^ To overcome the challenge associated with teaching medical students' sexual history taking, a multimodal approach has been found useful as it improves the students' performances during examinations.^26^

In Nigeria, the training of medical students on sexual health, including sexual history taking, is not domiciled in a particular specialty and thus remains an ad hoc topic covered by different specialties. The extent of the relevance to a particular specialty determines the degree of exposure that the students receive. This may account for why most students considered that they got enough exposure on sexual history taking during Obstetrics and Gynaecology (97%) and Urology (60%) postings.

Considering that most medical students in Nigeria eventually become General Practitioners, this study suggests a need to review the curriculum to ensure that they achieve sufficient proficiency in sexual history taking before graduation. It is thus recommended that further studies which will involve a large number of medical schools and students are carried out to explore this subject, including assessing the present modality of training on sexual health and the preferences of the medical students to design appropriate curriculum to improve the skills of medical students in sexual history taking.

### Limitations of the study

Being an online survey based on a specific assessment tool did not allow the students to air their opinions on how best they would have acquired proficiency in sexual history taking. Combining this method with a Focused Group Discussion may have yielded greater details on the students' perceptions on the topic. Also, the small number of medical schools and students in this study would affect the generalizability of the findings in Nigeria.

## Conclusion

This study showed that final year medical students are interested in and appreciated the importance of sexual history taking. Still, they are not well grounded in many aspects of the topic. Despite the importance of sexual health, many students did not have enough exposure and training on the topic while still in medical schools. Of note was the lack of exposure regarding observing doctors take sexual history or the students themselves taking a sexual history from real patients.
